# FtsK-Dependent Dimer Resolution on Multiple Chromosomes in the Pathogen *Vibrio cholerae*

**DOI:** 10.1371/journal.pgen.1000201

**Published:** 2008-09-26

**Authors:** Marie-Eve Val, Sean P. Kennedy, Meriem El Karoui, Laetitia Bonné, Fabien Chevalier, François-Xavier Barre

**Affiliations:** 1 CNRS, Centre de Génétique Moléculaire, UPR 2167, Gif-sur-Yvette, France; 2 Université Paris-Sud, Orsay, France; 3 Université Pierre et Marie Curie, Paris 6, Paris, France; 4 INRA, Unité des Bactéries Lactiques et Pathogènes Opportunistes, UR888, Jouy en Josas, France; National Cancer Institute, United States of America

## Abstract

Unlike most bacteria, *Vibrio cholerae* harbors two distinct, nonhomologous circular chromosomes (chromosome I and II). Many features of chromosome II are plasmid-like, which raised questions concerning its chromosomal nature. Plasmid replication and segregation are generally not coordinated with the bacterial cell cycle, further calling into question the mechanisms ensuring the synchronous management of chromosome I and II. Maintenance of circular replicons requires the resolution of dimers created by homologous recombination events. In *Escherichia coli*, chromosome dimers are resolved by the addition of a crossover at a specific site, *dif*, by two tyrosine recombinases, XerC and XerD. The process is coordinated with cell division through the activity of a DNA translocase, FtsK. Many *E. coli* plasmids also use XerCD for dimer resolution. However, the process is FtsK-independent. The two chromosomes of the *V. cholerae* N16961 strain carry divergent dimer resolution sites, *dif*1 and *dif*2. Here, we show that *V. cholerae* FtsK controls the addition of a crossover at *dif*1 and *dif*2 by a common pair of Xer recombinases. In addition, we show that specific DNA motifs dictate its orientation of translocation, the distribution of these motifs on chromosome I and chromosome II supporting the idea that FtsK translocation serves to bring together the resolution sites carried by a dimer at the time of cell division. Taken together, these results suggest that the same FtsK-dependent mechanism coordinates dimer resolution with cell division for each of the two *V. cholerae* chromosomes. Chromosome II dimer resolution thus stands as a bona fide chromosomal process.

## Author Summary

During proliferation, DNA synthesis, chromosome segregation, and cell division must be coordinated to ensure the stable inheritance of the genetic material. In eukaryotes, this is achieved by checkpoint mechanisms that delay certain steps until others are completed. No such temporal separation exists in bacteria, which can undergo overlapping replication cycles. The eukaryotic cell cycle is particularly well suited to the management of multiple chromosomes, with the same replication initiation and segregation machineries operating on all the chromosomes, while the bacterial cell cycle is linked to genomes of less complexity, most bacteria harboring a single chromosome. The discovery of bacteria harboring multiple circular chromosomes, such as *V. cholerae*, raised therefore a considerable interest for the mechanisms ensuring the synchronous management of different replicons. Here, we took advantage of our knowledge of chromosome dimer resolution, the only bacterial segregation process for which coordination with cell division is well understood, to investigate one of the mechanisms ensuring the synchronous management of the smaller, plasmid-like, and larger, chromosome-like, replicons of *V. cholerae*.

## Introduction

*Vibrio cholerae*, the causative agent of cholera, harbors two non-homologous circular chromosomes [1]. The majority of genes believed to be necessary for the basic life processes of *V. cholerae* are carried on the 2.96 Mbp chromosome I, whereas the 1.07 Mbp chromosome II only harbors a few essential genes [1]. The preferential transcription of genes from chromosome II during colon colonization [2] suggests that this genomic organization is important for pathogenicity. Likewise, other bacteria with multiple chromosomes can adopt several different life cycles [3], which led to the idea that multipartite genomes offer a selective advantage for the adaptation to very different environmental conditions.

Nevertheless, most bacteria harbor a single chromosome. In contrast, there is no apparent limit to the size and numbers of chromosomes harbored by eukaryotic cells. An important difference between bacteria and eukaryotes is that specific machineries appear to exist for the coordinated maintenance of each chromosome of a given bacterium, whereas eukaryotic cells possess a single global system for all chromosomes [4]–[12]. For instance, the two *V. cholerae* chromosomes harbor different partition systems [4],[7] and initiation of their replication is governed by different mechanisms [6],[8],[9]. In addition, many features of *V. cholerae* chromosome II, such as its partition system, are plasmid-like, which raised questions concerning its chromosomal nature [1],[10],[13]. Plasmid replication and segregation are generally not coordinated with the bacterial cell cycle [14], further raising questions on the mechanisms ensuring the synchronous management of chromosome I and II.

**Figure 1:**
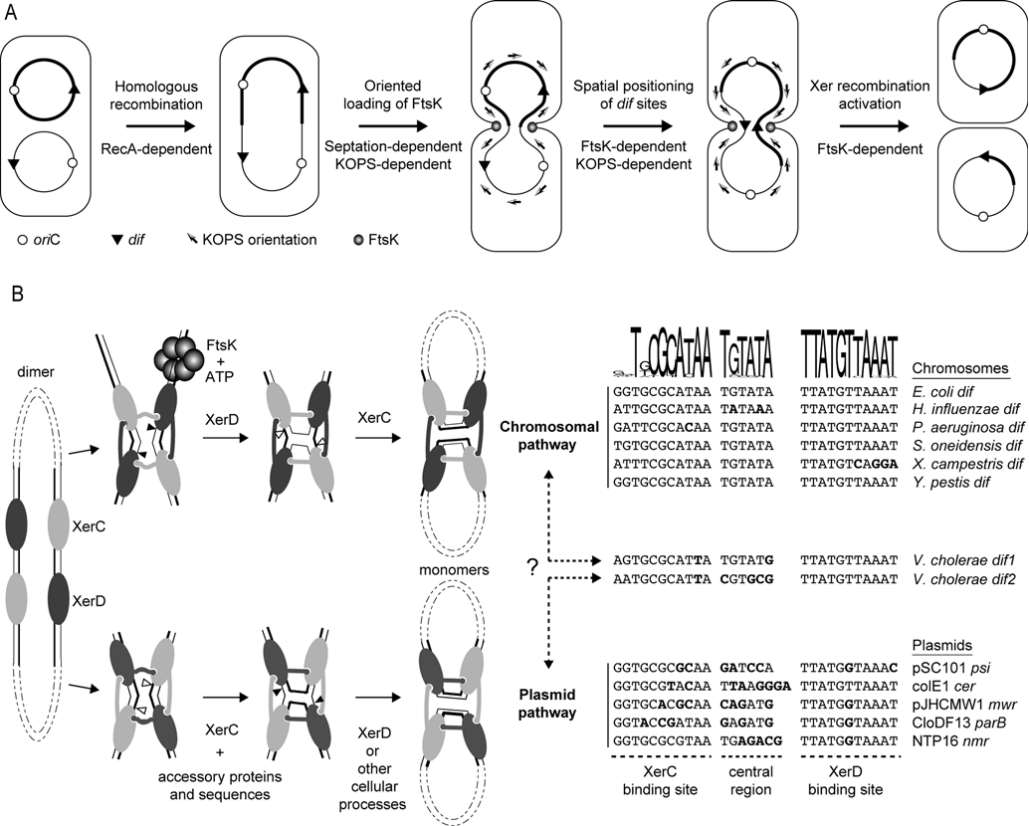
FtsK-dependent and FtsK-independent Xer recombination. A. Chromosome dimer formation and resolution in *E. coli*. The two homologous chromosomes are depicted by thick and thin lines, to allow for the visualization of crossovers. B. Link between the central region of XerCD-target sites (right) and the recombination pathway adopted at these sites (left). The XerCD-*dif* recombination complex is viewed from the C-terminal side of the recombinases, to show the C-terminal interactions of XerC and XerD. Strands cleaved by XerC and XerD in *E. coli* are shown with thick and thin lines, respectively. Positions of strand cleavages by *E. coli* XerC and XerD are indicated by white and black triangles, respectively. The WebLogo was generated using the alignment of putative *dif* sites from the larger chromosome of 27 γ-Proteobacteria (Text S1). The XerC-binding site, XerD-binding site and central region of *dif* ^Ec^ are indicated below the alignment.

A second major difference between bacteria and eukaryotes is intrinsic to the structure of chromosomes: in bacteria, chromosomes are generally covalently closed circular DNA molecules while they are linear in eukaryotes. DNA circularity can result in the formation of chromosome dimers by homologous recombination [15], which poses a barrier to the segregation of genetic information if they are not resolved before cell division (Figure 1A). Indeed, inactivation of chromosome dimer resolution (CDR) in *Escherichia coli* results in ∼15% cell death per generation under laboratory growth conditions [16], which corresponds to the estimated rate of chromosome dimers formed at each cell generation [17]. This prompted us to study how dimer resolution is achieved on each of the two *V. cholerae* chromosomes.

The mechanism of CDR was originally elucidated in *E. coli*. In this organism, it depends on the addition of a crossover at *dif*, a 28bp site located at the opposite of the origin of replication on the chromosome, by two related tyrosine recombinases, XerC and XerD (Figure 1A; see [18] for a review). In addition, CDR depends on two activities of a cell division protein, FtsK. First, FtsK functions as a DNA pump anchored in the septum [19],[20]. It loads on DNA trapped within the division septum due to dimer formation (Figure 1A). FtsK loading is oriented by specific DNA motifs, the KOPS, which dictates the orientation of translocation (Figure 1A; [21]). KOPS are skewed on the two replichores of the chromosome with *dif* located at the junction of their polarity [22],[23]. Thus, *dif* sites carried by a dimer are brought together by FtsK translocation (Figure 1A). Second, FtsK serves to activate recombination at *dif* via a direct interaction with XerD [24],[25]. *dif* contains two 11bp binding sites for XerC and XerD, separated by a central region at the outer boundary of which recombination occurs. The interaction between XerD and FtsK allows XerD to perform a first pair of strand exchanges [20], resulting in the formation of a Holliday junction (HJ). This HJ is converted to a crossover by a second pair of strand exchanges, which is catalyzed by XerC independently of FtsK (Figure 1B, chromosomal pathway). Thus, in *E. coli*, the requirement for FtsK to bring *dif* sites together and to activate the catalytic activity of XerD permits coordination of CDR with the last stage of cell division [26].

The *E. coli* pathway of CDR is not universal. For instance, *Streptococci* and *Lactococci* possess only a single tyrosine recombinase, XerS, for CDR [27]. Plasmid and viruses have also adopted different site-specific recombination systems to avoid multimerization of their genome. In *E. coli*, some of them depend on their own recombinases, such as phage P1, which encodes the Cre tyrosine recombinase [28], while others use the two Xer recombinases of their host [29],[30]. In the later case, XerC-catalysis initiates recombination independently of FtsK (Figure 1B, plasmid pathway, [18]). In this case, however, recombination requires ∼200bp of accessory sequences flanking the plasmid sites and which are bound by accessory proteins.

Orthologues of *E. coli xerC*, *xerD* and *ftsK* are readily identified on the larger chromosome of the *V. cholerae* strain N16961 (*xerC* ^Vc^, *xerD* ^Vc^, *ftsK* ^Vc^, Figure S1) whereas its second chromosome does not encode any site-specific recombination system that could be implicated in CDR apart from the superintegron integrase (IntIA, Figure S1). N16961 chromosome I and II both carry *dif*-like sequences, *dif*1 and *dif*2, which were originally identified as integration sites for the Cholera Toxin phage, CTXφ [31]. The weak filamentous phenotype of *V. cholerae* cells deleted for *xerC* or *dif*1 fits with a defect in CDR [31]. However, two features of the *V. cholerae* Xer recombination system, which could be linked to the co-existence of distinct, non-homologous chromosomes inside the same bacterium, were intriguing. First, *dif*2 differs from the *dif* consensus of γ-Proteobacteria by 5 bases, four of which belong to the central region (Figure 1B). Such a divergence is only found on plasmid sites, which, coupled with the other plasmid-like features of chromosome II, suggested that chromosome II dimer resolution might follow a plasmid pathway. Second, it was reported that the position of cleavage of XerD^Vc^ on *dif*1 might differ from the one of its *E. coli* orthologue on *dif* [32], even if *dif*1 differs from the *dif* consensus of γ-Proteobacteria by only 2 bases (Figure 1B), further raising questions on the exact mechanisms coordinating CDR of chromosome I and II with the cell cycle.

Here, we present the first formal study of CDR in *V. cholerae* and measure the rate of chromosome dimer formation on its two chromosomes under laboratory growth conditions. We show that the cell division protein FtsK^Vc^ is required for recombination by XerC^Vc^ and XerD^Vc^ at *dif*1 and *dif*2. In addition, we show that the activity of FtsK^Vc^ is directed by specific DNA motifs, which display the same skewed distribution on the two chromosomes, *dif*1 and *dif*2 being located at the junction of their polarity. Taken together, these results suggest that the same FtsK-dependent mechanism coordinates dimer resolution on each of the two *V. cholerae* chromosomes with cell division. Chromosome II dimer resolution thus stands as a bona fide chromosomal process.

## Results

### Chromosome Dimer Formation in *V. cholerae*

**Figure 2:**
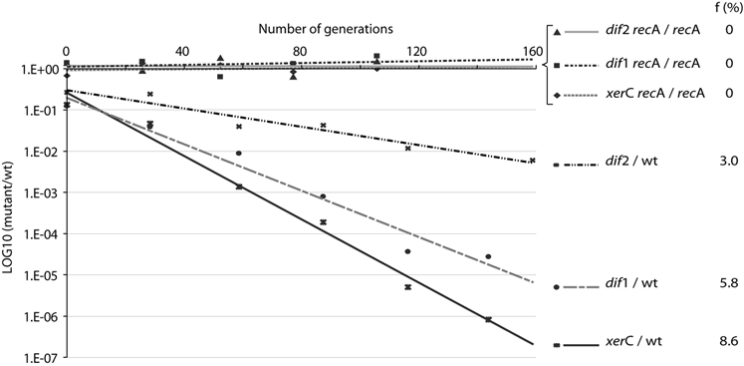
Growth competition of *V. cholerae* deficient in CDR strains against their parent. f: frequency of cells that the mutant strains fail to produce at each generation compared to their parent.

The growth of *V. cholerae* strains deficient in CDR was directly compared to the growth of their parental strain in competition experiments in rich media (Figure 2). These experiments revealed a defect of 5.8% and 3% per cell per generation for Δ*dif*1 and Δ*dif*2 cells, respectively, compared to their wild type counterparts. Since these growth defects were entirely suppressed in a *rec*A background (Figure 2), they directly reflect the rates of dimer formation on chromosome I and II, f_dimer_^Chr1^ and f_dimer_^Chr2^ (See Material and Methods). The 8.6% growth defect of *xer*C^Vc^ cells, which was also suppressed in a *rec*A background, reflects the total rate of chromosome dimer formation in *V. cholerae*, f_dimer_^Chr1+2^ (Figure 2). Interestingly, f_dimer_^Chr1+2^ equals 1−(1−f_dimer_^Chr1^)(1−f_dimer_^Chr2^), indicating that dimer formation on the two *V. cholerae* chromosomes is independent.

### In Vitro Cleavage by the *V. cholerae* recombinases on *dif*1 and *dif*2

**Figure 3:**
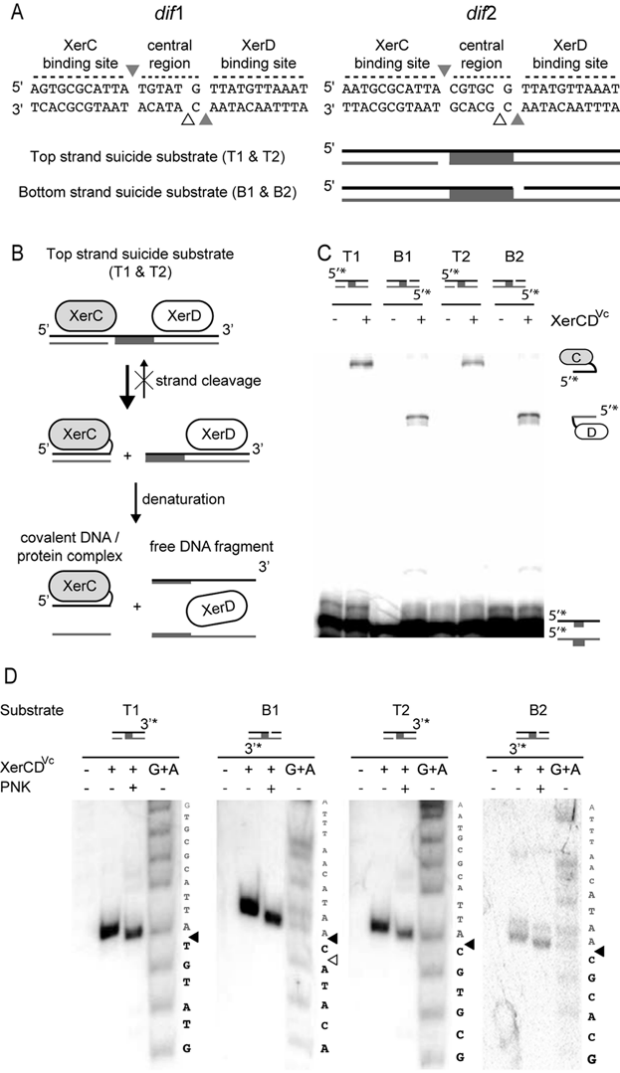
In vitro cleavage of *dif*1 and *dif*2 by the *V. cholerae* recombinases. A. Putative XerC^Vc^ and XerD^Vc^ cleavage sites on *dif*1 and *dif*2 and scheme of the suicide substrates used in this study. The top and bottom strands of *dif*1 and *dif*2 are depicted as black and grey strands. Their equivalents in *dif* ^Ec^ are cleaved by XerC^Ec^ and XerD^Ec^, respectively. Grey triangles further indicate the positions equivalent to these where XerC^Ec^ and XerD^Ec^ cleave *dif* ^Ec^. A white triangle indicates the XerD^Vc^-cleavage position reported for *dif*1 [32]. Top and bottom strand suicide substrates contain a nick opposite the position expected to be cleaved by XerC^Vc^ and XerDVc if the *E. coli* paradigm is followed, respectively. T1, B1, T2, B2: suicide substrates on *dif*1 and *dif*2, respectively. B. Scheme of a XerC-suicide cleavage reaction. C. Covalent complex formation by MBPXerC^Vc^ and XerD^Vc^ on suicide substrates. Schemes of substrates and products are shown on the top and on the right of the gel, respectively. Suicide substrates were labeled on the 5′ side of the continuous strand, as indicated (5′*). D. Cleavage sites of XerC^Vc^ and XerD^Vc^ on *dif*1 and *dif*2. Schemes of substrates are shown on the top of the gels. Suicide substrates were labeled on the 3′ side of the continuous strand, as indicated (3′*). PNK: phosphorylation with T4 polynucleotide kinase; G+A: chemical cleavage ladder. Sequences resulting from the chemical cleavage are indicated beside the gels. Bases of the central region and of the XerCD-binding sites are indicated in black and grey, respectively. The deduced cleavage points are indicated by black triangles.

Recombinase-mediated strand cleavage can be assayed *in vitro* using suicide substrates that contain a nick opposite of the position of cleavage (Figure 3A). Cleavage of the continuous strand of a suicide substrate generates a double strand break that prevents re-ligation (Figure 3B). This leads to (i) the accumulation of covalent protein/DNA complexes between the attacking recombinase and the 5′-end fragment of the continuous strand and (ii) the accumulation of free 3′-end fragments of the continuous strand (Figure 3B). XerC^Ec^ and XerD^Ec^ each cleave a specific strand on *dif* ^Ec^. The strand cleaved by XerC^Ec^ is termed Top strand. The strand cleaved by XerD^Ec^ is termed Bottom strand. Following this convention, suicide substrates in which the continuous strand is expected to be cleaved by XerC^Vc^ are called Top strand suicide substrates and suicide substrates in which the continuous strand is expected to be cleaved by XerD^Vc^ are called Bottom strand suicide substrates (Figure 3A).

Labeling the 5′-end of the continuous strand of suicide substrates allows the detection of covalent recombinase/DNA complexes (Figure 3C). The molecular weight of XerC^Vc^ and XerD^Vc^ being very similar, we used a maltose binding protein fusion of XerC^Vc^ (MBPXerC^Vc^) in conjunction with XerD^Vc^ to avoid any confusion between the two possible covalent complexes. For both *dif*1 and *dif*2, MBPXerC^Vc^-DNA covalent complexes accumulated when Top strand suicide substrates were used (Figure 3C, T1 and T2, respectively), indicating that XerC^Vc^ cleaves the Top strands of *dif*1 and *dif*2. Furthermore, XerD^Vc^-DNA covalent complexes accumulated when Bottom strand suicide substrates were used (Figure 3C, B1 and B2), indicating that XerD cleaves the bottom strands of *dif*1 and *dif*2.

The position of cleavage of XerC^Vc^ and XerD^Vc^ were then determined by comparing of the length of the free DNA fragments liberated by recombinase cleavage to a ladder obtained by chemical cleavage at purine bases of the suicide substrates (Figure 3D). To this aim, the continuous strands of the suicide substrates were labeled on their 3′ end. Cleavage by tyrosine recombinases generates a 5′OH DNA extremity whereas chemical cleavage leaves a 5′ phosphate. As a consequence, the free DNA fragments had to be first phosphorylated by kinase treatment (Figure 3D, PNK) in order to be compared with the chemical cleavage ladder (Figure 3D, G+A). We thus found that XerC^Vc^ and XerD^Vc^ cleave DNA at the junction between their respective binding site and the central region of *dif*1 and *dif*2 (Figure 3D, black arrows).

### FtsK-Dependent Recombination at *dif*1 and *dif*2

**Figure 4:**
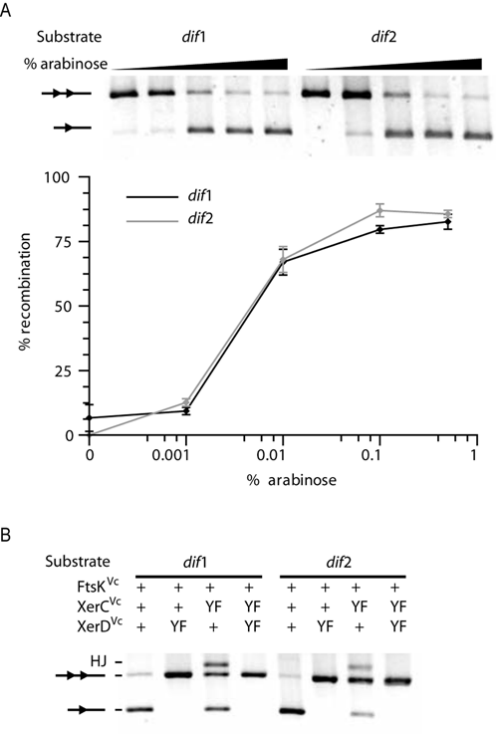
FtsK^Vc^-dependent recombination at *dif*1 and *dif*2. A. Reconstitution of *V. cholerae* Xer recombination at plasmid-borne *dif*1 and *dif*2 sites in *E. coli* cells. Top panel: gel showing a typical result. A scheme of the substrate and product bands is shown beside the gel. *dif* sites are represented by triangles. Bottom panel: quantification plot displaying the mean and standard deviations of at least three independent experiments. B. Recombination by wild-type (+) and catalytically inactive (YF) recombinases. HJ: HJ intermediate.

Analysis of the DNA sequence immediately upstream and downstream of *dif*1 and *dif*2 in different Vibrio species did not reveal any conserved motifs that could serve to bind accessory proteins (data not shown). FtsK^Vc^ was thus left as the most likely candidate for activation of Xer recombination at both sites. To test this possibility, we reconstituted the *V. cholerae* Xer system in *E. coli* cells deleted for their natural FtsK/XerCD system. We used a *xerC* and *xerD E. coli* strain, which was also *fts*K_C_^−^. This strain produces only the N-terminal domain of FtsK^Ec^, essential for viability [33], but lacks production of the C-terminal domain of FtsK^Ec^, which is necessary for recombination at *dif* ^Ec^ [34]. XerC^Vc^ was expressed in conjunction with XerD^Vc^ from the chromosomal *E. coli xerC* promoter. The production of FtsK^Vc^ was controlled by placing the full length *fts*K^Vc^ ORF under an arabinose-inducible promoter on a high-copy number plasmid. A low-copy plasmid carrying two recombination sites in direct repeats was used as a reporter. Recombination between the two repeated sites results in the excision of the intervening DNA, which can be monitored by agarose gel electrophoresis. For both *dif*1 and *dif*2, the amount of recombination correlated with the amount of arabinose used for induction, indicating that Xer recombination at *dif*1 and *dif*2 depends on FtsK^Vc^ (Figure 4A).

To determine the order of the strand exchanges in the recombination reactions, we monitored plasmid recombination in a set of four strains encoding either wild-type XerC^Vc^ and XerD^Vc^ or the XerC_YF_^Vc^ and XerD_YF_^Vc^ mutants, in which the catalytic tyrosine is replaced by a phenylalanine (Figure 4B). For both *dif*1 and *dif*2, no resolution product or HJ intermediate were detected in XerD_YF_^Vc^ cells (Figure 4B, lane 2, 4, 6 and 8). In contrast, we could detect the accumulation of a HJ intermediate in XerC_YF_^Vc^ XerD^Vc^ cells (Figure 4B, lane 3 and 7), indicating that XerD^Vc^ mediates the first pair of strand exchanges during both *dif*1 and *dif*2-recombination. Recombination products were likely still observed in XerC_YF_^Vc^ XerD^Vc^ cells since other cellular processes than Xer recombination are capable of resolving HJs [18]. However, the amount of product was considerably decreased, indicating that intermediate HJs are preferentially resolved to crossovers by the action of XerC^Vc^.

All together, these results indicate that FtsK^Vc^ activates recombination at *dif*1 and *dif*2 by promoting the exchange of a first pair of strands by XerD^Vc^.

### Species-Specificity in Xer Recombination Activation

**Figure 5:**
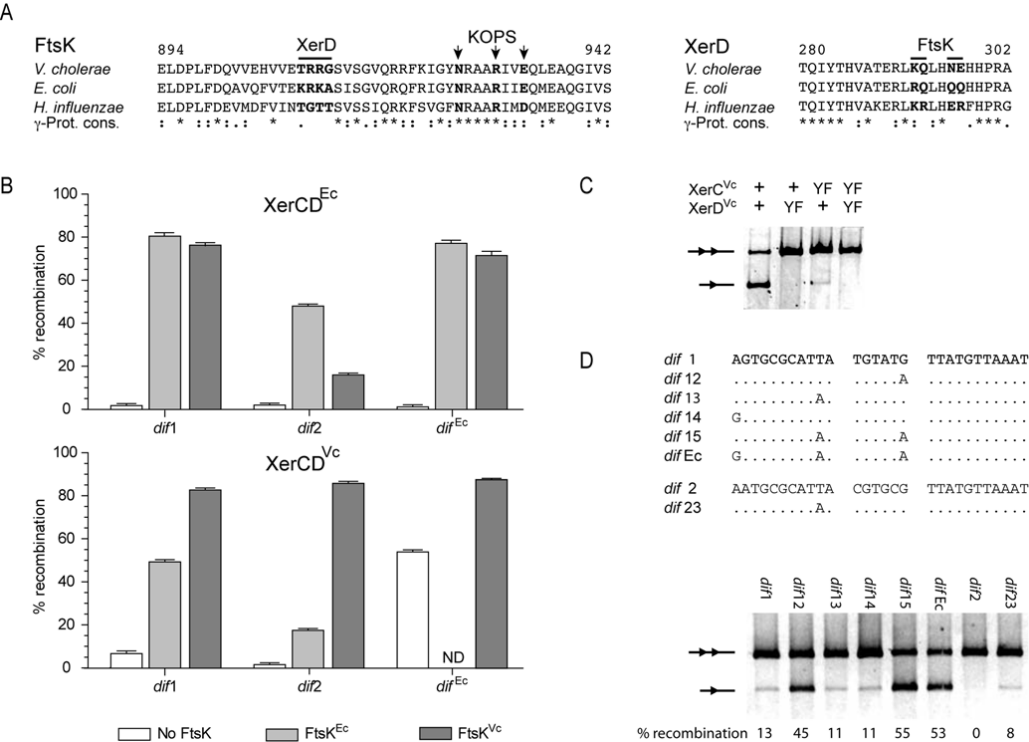
Species specificity in Xer recombination. A. Amino acid residue conservation in the γ-domain of FtsK and in the C-terminal tail of XerD. Numbers indicate the position of the first and of the last residues of the alignments in the amino acid sequence of the *V. cholerae* proteins. Positions of full conservation and of strong or weaker groups of conservation are indicated by stars, semi-colons or dots, respectively, following the Clustal 1.83 scheme. Black bars and vertical arrows indicate residues implicated in FtsK-XerD interaction and in KOPS recognition in *E. coli*, respectively. B. Species-specificity in Xer recombination on plasmid-borne *dif*1, *dif*2 and *dif* ^Ec^ sites. The mean and standard deviation of at least three independent experiments are plotted. ND: not determined. C. FtsK-independent recombination at *dif* ^Ec^ by wild-type (+) and catalytically inactive (YF) *V. cholerae* recombinases. D. FtsK-independent recombination by the *V. cholerae* recombinases on hybrid *dif* sites.

Several residues implicated in the interaction between *E. coli* XerD and FtsK have been mapped [24],[25]. These residues are not entirely conserved between the *V. cholerae* and *E. coli* proteins (Figure 5A), suggesting that the interactions between the translocase and the recombinases might be specific in these two species. Nevertheless, both FtsK^Ec^ and FtsK^Vc^ could activate recombination by XerCD^Ec^ and XerCD^Vc^ at *dif* ^Ec^, *dif*1 and *dif*2 (Figure 5B). However, the efficiency of recombination varied for each site and for each pairing of translocase/recombinases. XerCD^Ec^-recombination at *dif* ^Ec^ and *dif*1 reached 80% of efficiency whether FtsK^Ec^ or FtsK^Vc^ were produced (Figure 5B, XerCD^Ec^, *dif*1 and *dif* ^Ec^). In contrast, XerCD^Ec^-recombination at *dif*2 was more efficient when activated by FtsK^Ec^ than FtsK^Vc^ (Figure 5B, XerCD^Ec^, *dif*2). In addition, it did not reach 80% efficiency, even in the presence of the cognate partner translocase, FtsK^Ec^. XerCD^Vc^-recombination at *dif* ^Ec^, *dif*1 and *dif*2 reached 80% of efficiency (Figure 5B, XerCD^Vc^). However, this required the presence of FtsK^Vc^. XerCD^Vc^-recombination at *dif*2 even fell below 20% when activated by FtsK^Ec^. Thus, the effect of species-specificity is more pronounced on *dif*2 than on *dif*1.

### Importance of the Sequence of the Resolution Sites for the Stringent Control of Xer Recombination

We noticed that the *V. cholerae* recombinases could promote recombination between *dif*1 sites in the absence of FtsK production, albeit to a very low level (Figure 5B, XerCD^Vc^, *dif*1, No FtsK). This was further exemplified on *dif* ^Ec^ substrates, in which 53% of recombination was observed without FtsK expression (Figure 5B, XerCD^Vc^, *dif* ^Ec^, No FtsK). Resolution products were detected in the absence of XerC catalysis (Figure 5C, XerC_YF_^Vc^ strains) but not in the absence of XerD catalysis (Figure 5C, XerD_YF_^Vc^ strains), signifying that XerD^Vc^ catalysis initiated recombination. *dif*1 differs from the γ-Proteobacteria consensus by only 2 bp, the substitution of A^17^ by G and the substitution of A^10^ by T (Figure 5D). We therefore analyzed FtsK-independent XerCD^Vc^ recombination at hybrid sites between *dif*, *dif*1 and *dif*2 to identify residues important for the above observation (Figure 5D). A site carrying the single [G-A]^17^ substitution promoted a much higher level of FtsK-independent recombination (*dif*12), while recombination at sites carrying the [T-A]^10^ and [G-A]^1^ substitutions was not altered (*dif*13 and *dif*14). However, the cumulative substitutions of [G-A]^17^ and [T-A]^10^ increased FtsK-independent recombination to a level equivalent to *dif* ^Ec^-recombination (*dif*15). In addition, when T^10^ was altered to A in *dif*2, we observed a faint recombination product (*dif*23), which was significant since FtsK-independent recombination was never observed at *dif*2. Thus, G^17^ in the central region of *dif*1 and T^10^ in the XerC-binding site of *dif*1 and *dif*2 appear to have an important role in maintaining Xer recombination under the tight control of FtsK in *V. cholerae*.

### *V. cholerae* FtsK Orienting Polar Sequences

**Figure 6:**
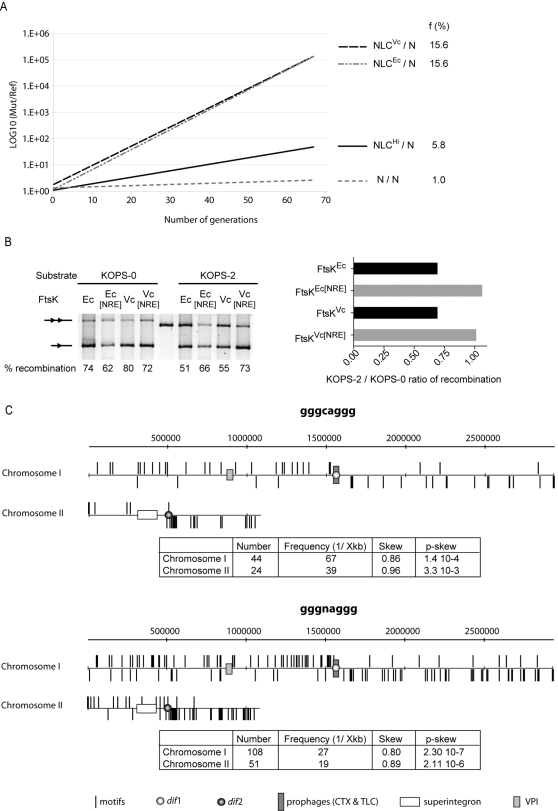
* V. cholerae* FtsK Orienting Polar Sequences. A. Growth competition of *E. coli* cells encoding FtsK hybrids. N: cells carrying a complete deletion of the C-terminal domain and linker region of FtsK^Ec^; NLC^Ec^: cells carrying full length FtsK^Ec^; NLC^Vc^ and NLC^Hi^: cells in which the C-terminal domain FtsK^Ec^ was replaced by the one of FtsK^Vc^ and FtsK^Hi^, respectively. f: frequency of cells that the parental N strain fails to produce at each generation compared to the FtsK hybrids. B. 5′-GGGCAGGG-3′ inhibits recombination activation by FtsK^Vc^. Plasmid recombination at *E. coli dif* by XerCD^Ec^ was induced with 0.5% arabinose. Ec[NRE]: FtsK_50C_^Ec^[NRE]; Vc[NRE]: FtsK^Vc^[NRE]. KOPS-0: substrate without GGGCAGGG sequences; KOPS-2: substrate with triple overlapping GGGCAGGG sequences in the non-permissive orientation on both sides of the two *dif* sites. C. Scheme of the two *V. cholerae* chromosomes showing the distributions of the GGGCAGGG and GGGNAGGG motifs. Upper bars: motifs found in the leading strand; Lower bars: motifs found in the lagging strand. Number, frequency, skew and skew significance (*p*-skew) are indicated for each motif. Recently acquired genomic regions are indicated (superintegron, CTX and TLC prophages and the Vibrio Pathogenicity Island VPI).

We next investigated if FtsK^Vc^ could serve to bring together the CDR sites carried by dimers of chromosome I or by dimers of chromosome II. Several key residues implicated in KOPS recognition have been identified in the γ domain of FtsK^Ec^ (Figure 5A; N1296; R1300; E1303; [35]). The conservation of these residues in FtsK^Vc^ suggested that it could recognize the same motifs (Figure 5A; N926; R930; E933). If this was indeed the case, replacing the C-terminal domain of FtsK^Ec^ with the one of FtsK^Vc^ should completely rescue CDR in *E. coli* cells since FtsK^Vc^ fully activates recombination by XerCD^Ec^ at *dif* ^Ec^ (Figure 5B, XerCD^Ec^, *dif* ^Ec^, FtsK^Vc^). Indeed, the fitness of such cells equaled the fitness of wild-type *E. coli* cells in growth competition experiments (Figure 6A, NLC^Vc^ and NLC^Ec^), in contrast to cells only expressing the N-terminal domain of FtsK^Ec^ or a fusion with the C-terminal domain of *H. influenzae* FtsK (Figure 6A, N and NLC^Hi^).

To test for the ability of FtsK^Vc^ to specifically recognize one of the *E. coli* KOPS motifs, we compared the efficiency with which it activates *E. coli* Xer recombination between plasmid-borne *dif^Ec^* sites flanked or not by the 5′-GGGCAGGG-3′ motif in an orientation that should prevent it from translocating towards *dif^Ec^* (Figure 6B, KOPS-2 and KOPS-0, respectively). Here we observed that the efficiency of recombination dropped significantly on KOPS-2 when FtsK^Ec^ or FtsK^Vc^ were used as activators (Figure 6B, FtsK^Ec^ and FtsK^Vc^). We then engineered an allele of *ftsK^Vc^* carrying identical mutations to the one shown to abrogate KOPS recognition in FtsK^Ec^ [35]. No difference in recombination efficiency was noticeable between KOPS-0 and KOPS-2 when using this allele or its *E. coli* homologue (Figure 6B; FtsK_50C_^Ec^[N1296A; R1300A; E1303A] and FtsK^Vc^[N926A; R930A; E933A]). We conclude that FtsK^Vc^ directly recognizes the GGGCAGGG motif and that recognition engages amino acids N926, R930 and E933.

We decided therefore to analyze the skew and frequency of the GGGCAGGG motif on chromosome I and II. GGGCAGGG is highly polarized on both chromosomes with statistically significant skews (Figure 6C). On chromosome I, the skew switches precisely at *dif*1 whereas on chromosome II one motif is present on the reverse orientation a few kb before *dif*2 (Figure 6C). However, it has been shown in *E. coli* that a single non-permissive KOPS motif in the vicinity of *dif* is not sufficient to impair recombination [23]. The frequency of GGGCAGGG is low on both *V. cholerae* chromosomes (Figure 6C), suggesting that this motif is not sufficient by itself to provide polar orientation of FtsK^Vc^. We therefore analyzed the distribution of all octamers motif families with one degenerated position on both chromosomes. We ranked potential candidates according to their skew significance keeping only families that had a skew of at least 80% and a frequency of at least once every 30 kb. Only one family (GGGNAGGG) was among the 10 best candidates of both chromosomes. This family is highly skewed, frequent (Figure 6C) and contains the experimentally active GGGCAGGG motif. Taken together, these results suggest that the GGGNAGGG motifs might function as KOPS in *V. cholerae*.

## Discussion

### A Common Cell Division-Coordination Mechanism for Dimer Resolution of the Two *V. cholerae* Chromosomes

The strand exchanges catalyzed by XerC^Vc^ and XerD^Vc^ occur at the junction between their respective binding site and the central region of *dif*1 and *dif*2, as previously reported for the *E. coli* recombinases on *dif* (Figure 3). FtsK^Vc^ promotes recombination at both sites by activating a first pair of strand exchanges mediated by XerD^Vc^ (Figure 4), thanks to a species-specific interaction with the recombinases (Figure 5). In addition, GGGNAGGG motifs seem to function as FtsK^Vc^-Orienting Polar Sequences, their frequency and distribution on the two *V. cholerae* chromosomes suggesting that the FtsK^Vc^-translocase activity helps bring CDR sites together when dimers are formed on chromosome I or on chromosome II (Figure 6). We conclude that the same FtsK-dependent mechanism controls dimer resolution on each of the two *V. cholerae* chromosomes. We have previously shown in *E. coli* that the requirement for FtsK to activate Xer recombination delays CDR to the time of septum closure [26], which is likely to also hold true in *V. cholerae*. Thus, the study of CDR provides the first example of a cell cycle coordination mechanism shared by the two *V. cholerae* chromosomes, which is similar to the way chromosomal maintenance processes are coordinated within the cell cycle of eukaryotes.

### Dimer Formation Is Linked to Replicon Size

Many bacteria harbor multiple chromosomes, which seems an important determinant of their individual life styles. A few bacterial species harbor linear replicons in addition to circular, such as *Agrobacterium tumefaciens* and the *Borrelia* species [3]. In the vast majority of cases, however, the multiple chromosomes harbored within a bacterium are circular. Maintenance of circular replicons requires the resolution of dimers created by homologous recombination events. In *V. cholerae*, 5.8% of dimers per cell per generation are formed on the 2.96 Mbp chromosome I and 3% of dimers are created on the 1.07 Mbp chromosome II (Figure 2). Under similar growth conditions, 15.6% of dimers are generated on the 4.6Mbp *E. coli* chromosome (Figure 6). These results suggest that dimer formation increases with replicon size, possibly reaching a theoretical upper limit of 50% for very large replicons. In addition, the rate of dimer formation seems to vary exponentially with replicon size for small replicons. Based on this hypothesis, the frequency of chromosome dimer formation in *V. cholerae* would be 11% per cell generation if it carried a single circular chromosome of 4.03Mbp. Instead, we measured a total rate of 8.6% for the two chromosomes (Figure 2). Thus, the particular genomic organization of the Vibrios seems to minimize chances for chromosome dimer formation, which is theoretically beneficial.

### Generalization to Other Bacteria with Multiple Chromosomes

**Figure 7:**
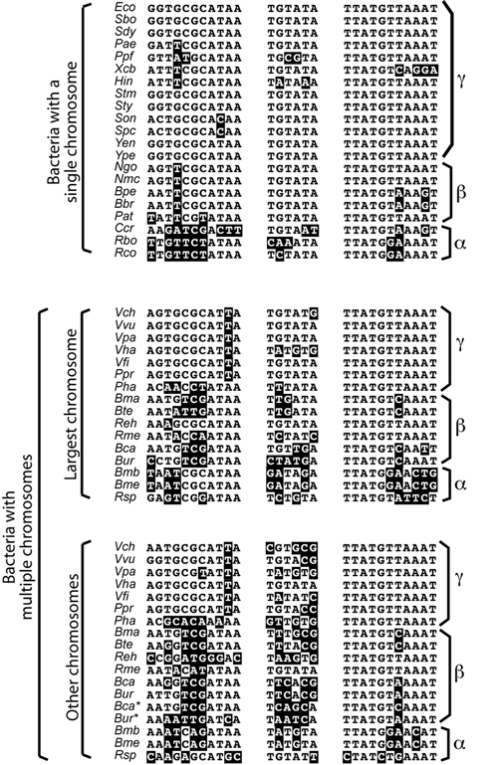
The non-homologous chromosomes of Proteobacteria with multipartite genomes carry divergent *dif* sites. Alignment of the chromosome dimer resolution sites of a few Proteobacteria harboring a single or multiple chromosomes. Bases identical to the γ-Proteobacteria *dif* consensus are shaded in black. Species abbreviations follow the KEGG convention.

Putative *dif* sites were readily identified on each of the two chromosomes harbored by 7 additional γ-Proteobacteria (Figure 7 and Figure S2). To determine *dif* sites in β- and α-Proteobacteria, we generated a profile Hidden Markov Model (HMM) based on the alignment of the putative CDR sites found in the larger chromosome of 27 γ-Proteobacteria using the program HHMER. We then compared each sequence by hand to ensure the proper 6 bp spacing between the putative XerC and XerD binding sites. Putative *dif* sites were thus identified on each of the multiple chromosomes harbored by 10 β-Proteobacteria species and 5 α-Proteobacteria species (Figure 7 and Figure S3 and S4). A single pair of recombinases orthologous to XerC and XerD was found in each of the 22 additional γ-, β- and α-Proteobacteria harboring multiple chromosomes, suggesting that a single pair of recombinases ensures dimer resolution of each of their non-homologous chromosomes. FtsK orthologues were also found. In addition, putative *dif* sites fell within 10 kb of the GC-skew inflection point (data not shown), suggesting that dimer resolution is under the control of an FtsK-like homologue in all these species. Thus, the adoption of an FtsK-dependent dimer resolution system could be a key evolutionary step in the maintenance of large circular replicons.

### Tuning of *V. cholerae* CDR to Achieve Efficient Recombination on Divergent *dif* Sites

The sequence of Xer target sites, and especially of their central region, is a crucial determinant in the outcome of recombination [36],[37]. Indeed, the central region of *dif* sites found in Proteobacteria with a single chromosome showed a high degree of conservation, most β- and γ-Proteobacteria harboring a ‘canonical’ 5′-TGTATA-3′ motif (Figure 7 and Figure S2 and S3), suggesting that there is a selective pressure on the sequence of the *dif* central region. This is further illustrated by the lower recombination efficiency of the *E. coli* system on *dif*2 compared to *dif*1 (Figure 5). In this regard, the *V. cholerae* Xer recombination system is remarkable since identical recombination efficiencies were obtained with the same pair of recombinases on *dif*1 and *dif*2 (Figure 4 and 5). However, XerCD^Vc^-mediated recombination at *dif*2 required a tighter interaction between the recombinases and their partner translocase than at *dif*1, since FtsK^Ec^ promoted 50% of recombination at *dif*1 but less than 20% at *dif*2 (Figure 5B, XerCD^Vc^, FtsK^Ec^). In addition, a few alterations in the sequence of *dif*1 and *dif*2 decreased the stringency of the control exerted by FtsK^Vc^ (Figure 5D), highlighting the extremely fine tuning of the different components of the *V. cholerae* CDR system.

### Non-Homologous Chromosomes Carry d*if* Sites with Divergent Central Regions

We observed that in Proteobacteria with multiple chromosomes, the central regions of *dif* sites from non-homologous chromosomes are divergent, as in *V. cholerae* (Figure 7 and Figure S2, S3, S4). A single exception was found in *Burkholderia xenovarans*, in which two of the three chromosomes of the bacterium harbor a resolution site with an identical central region. We reasoned therefore that some selective pressure imposes the divergence of the central regions of CDR sites carried by the different, non-homologous chromosomes of bacteria with multipartite genomes, which competes with the selective pressure for *dif* central regions to adopt the preferential 5′-TGTATA-3′ motif. Indeed, the presence of *dif* sites with identical central regions on two non-homologous chromosomes could lead to the formation of chromosome fusions by Xer recombination, which would disrupt the selective advantage brought by the multipartite genomic organization. In support of this hypothesis, preliminary experiments indicate that harmonization of the two *V. cholerae dif* sites leads to chromosomal fusions (Val and Barre, unpublished observations). We are currently investigating how these fusions are formed and the consequences of harboring identical *dif* sites on separate chromosomes.

## Materials and Methods

### Strains, Plasmids, and Media

All growth experiments were done in LB-Lennox. Strains and plasmids are listed in Text S1. Briefly, *V. cholerae* strains were derived from N16961 [1] by allele exchange using pDS132 derivatives [38] and *E. coli* β2163 as a donor strain [39]. *E. coli* strains used for *in vivo* plasmid resolution assays and for growth competition were engineered as previously described in [25],[40]. Mutations were confirmed by PCR and sequencing.

### Growth Competition Assay

For growth competitions, *E. coli* cells were grown at 37°C with a 1000× dilution in fresh media every 12h [40]. Because of their higher growth rate, *V. cholerae* cells were grown at 30°C with a 10000× dilution every 12h. The numbers of CFU of mutated and parental cells in the cultures were determined by plating on cognate antibiotic plates every 12 or 24h, depending on the mutant growth defect. These numbers were used to calculate the number of generation of the parent cells between each time points and the CFU ratio of mutated versus parent cells at each time point. This ratio varies exponentially with the number of generations. The proportion of cells that the mutant strain fails to produce at each doubling time of its parent is deduced from the coefficient of this exponential. This ratio is a good estimation of the rate of dimer formation (Text S1).

### In Vitro Xer Assays

*V. cholerae* MBP-XerD and MBP-XerC recombinases were purified using nickel, amylose and heparin columns. The MBP tag was removed by thrombin digestion. *dif*1 and *dif*2 synthetic suicide substrates (Text S1) were obtained by annealing synthetic oligonucleotides purified by PAGE. 5′-end labeling of oligonucleotides was performed using T4 DNA polynucleotide kinase and [^32^P] γ-ATP and 3′end labeling using terminal transferase and [^32^P] α-ddATP. Reactions were performed in 20 mM Tris-HCl (pH 7.5), 50 mM NaCl, 0.1mM EDTA, 1 μg/ml of BSA, 40% glycerol and 0.2 pmol of radiolabeled probe for 2 hours at 37°C. Covalent complexes were analyzed by 12% SDS-PAGE and cleavage sites by 12% urea-PAGE. Radioactivity was detected on a STORM (GE Healthcare).

### In Vivo Plasmid Resolution Assays

*E. coli* cells were transformed with the FtsK expression vector and then with the Xer recombination reporter plasmid, as described in [25]. 10 transformant colonies were pooled in 1 ml of LB, diluted 100× in LB and grown to 0.6 OD at 37°C. Cells were then grown for an extra 2 hours at 37°C in the presence of 0.5% arabinose to induce FtsK production, unless otherwise indicated. Plasmid DNA was hydrolyzed with *Nde*I (single cutter). Recombination efficiency was computed as the amount of replicative product over the sum of the amount of substrate and of replicative product, which were separated by agarose gel electrophoresis and detected with SybrGreen staining using a LAS-3000 (Fuji Life Science).

### Bioinformatics Analysis of Motifs Distribution

Leading strands were defined as the DNA strand reported in Genbank files downstream of the replication origin up to the terminus and the reverse complement strand from the terminus to the origin. The terminus position was chosen as the first nucleotide of the CDR site. Skew statistical significance was assessed by calculating the probability that the observed skew occurred by chance taking into account the fact that G-rich motifs are likely to be more frequent on the leading strand because of GC skew, as previously described [41]. Analysis on chromosome II was performed on a chimeric chromosome where the superintegron has been removed because this element carries more than 100 repetitions of the *att*C integration site, which hides the signal provided by octamer motifs.

## Supporting Information

Figure S1 XerCD and FtsK tree.
(2.80 MB TIF)

Figure S2 dif sites in gamma-Proteobacteria.
(6.03 MB TIF)

Figure S3 dif sites in beta-Proteobacteria.
(5.50 MB TIF)

Figure S4 dif sites in alpha-Proteobacteria.
(3.18 MB TIF)

Text S1 Supplementary methods.
(0.12 MB DOC)
