# Supplementary figures and images for: FtsK-Dependent Dimer Resolution on Multiple Chromosomes in the Pathogen Vibrio cholerae
Source: PLoS Genet. 2008 Sep 26;4(9):e1000201. doi: 10.1371/journal.pgen.1000201 (PMC2533119; doi:10.1371/journal.pgen.1000201)

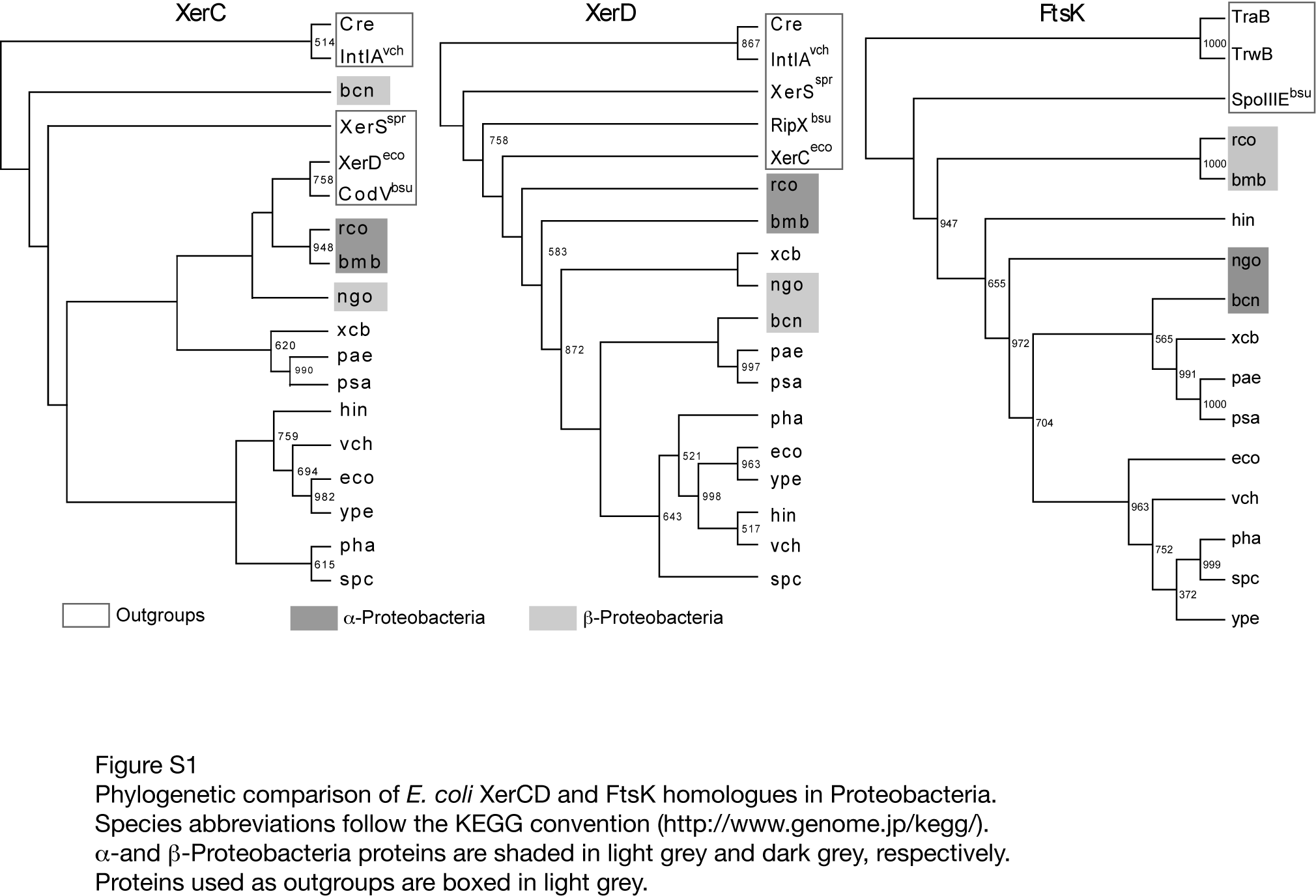

Supplement: Figure S1 — XerCD and FtsK tree. (2.80 MB TIF) [file pgen.1000201.s001.tif]

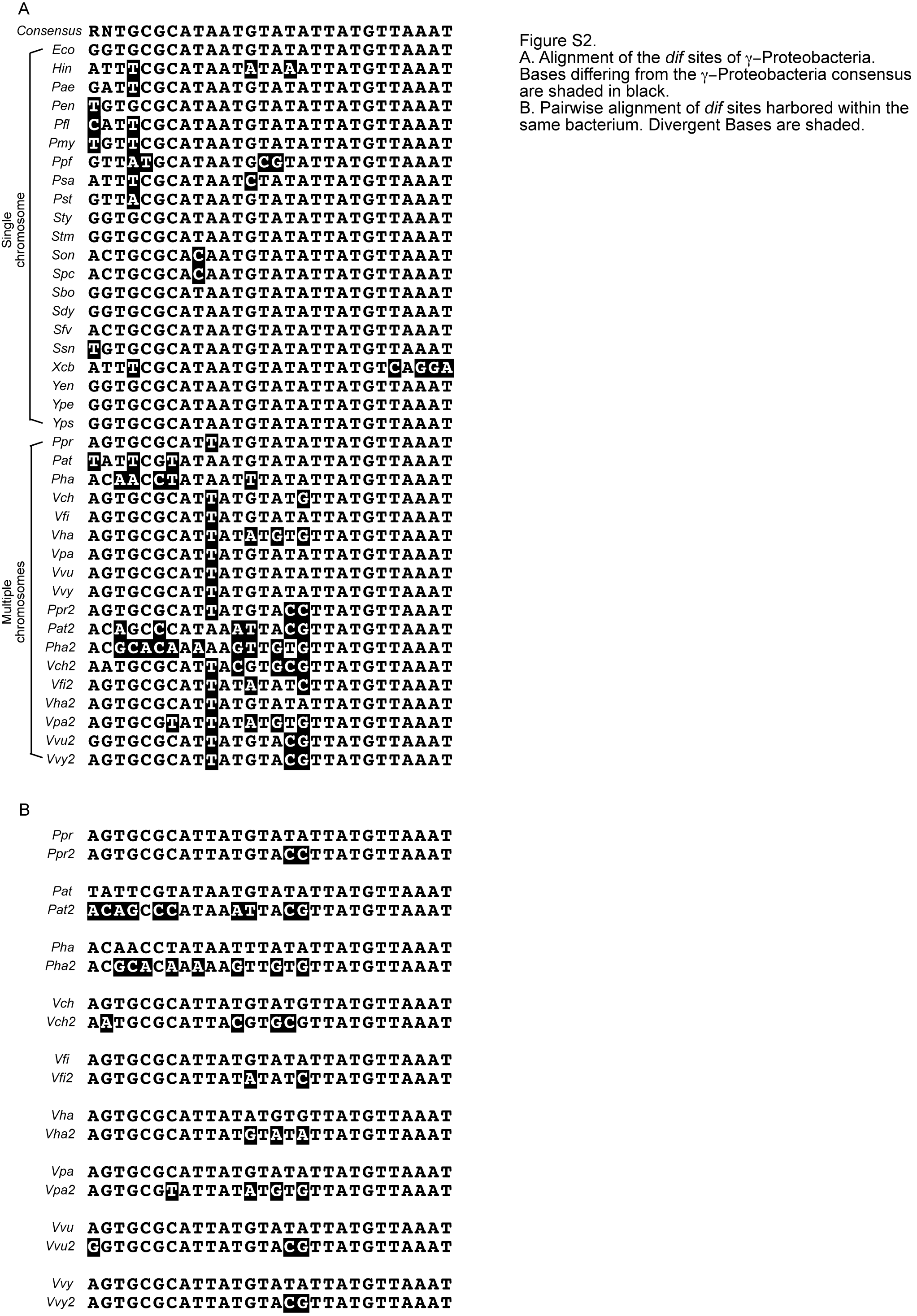

Supplement: Figure S2 — dif sites in gamma-Proteobacteria. (6.03 MB TIF) [file pgen.1000201.s002.tif]

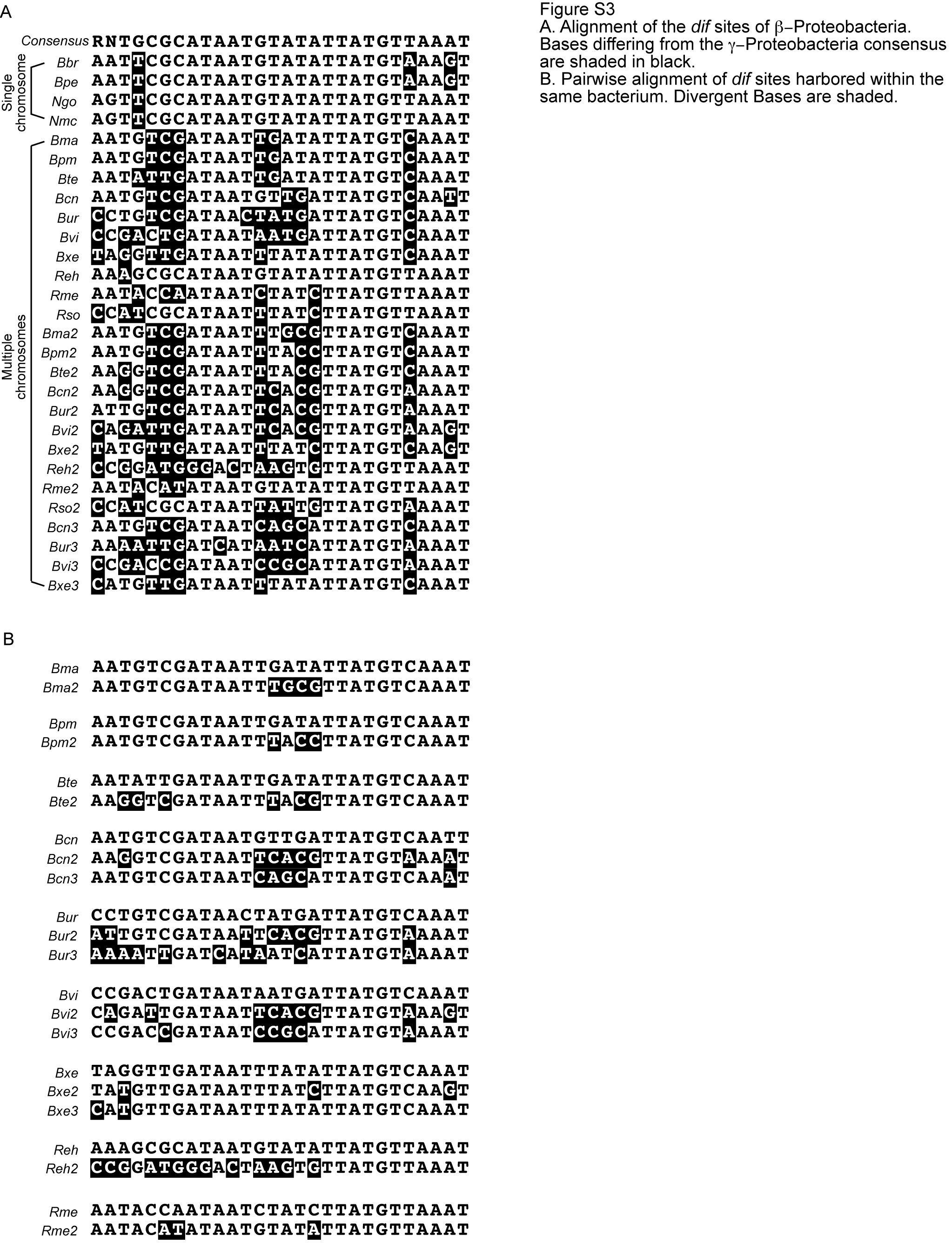

Supplement: Figure S3 — dif sites in beta-Proteobacteria. (5.50 MB TIF) [file pgen.1000201.s003.tif]

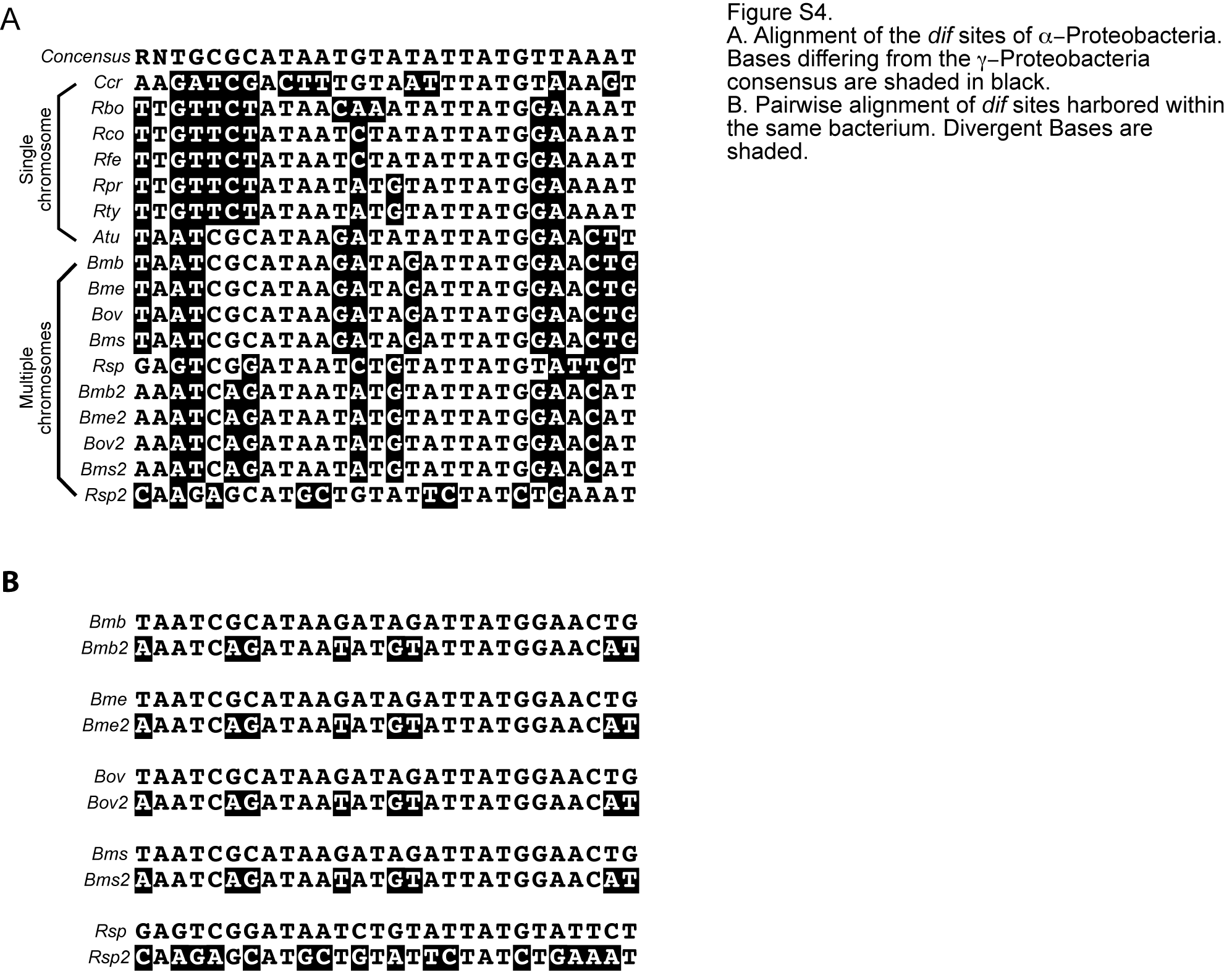

Supplement: Figure S4 — dif sites in alpha-Proteobacteria. (3.18 MB TIF) [file pgen.1000201.s004.tif]
