# Supplementary material for: FtsK-Dependent Dimer Resolution on Multiple Chromosomes in the Pathogen Vibrio cholerae
Source: PLoS Genet. 2008 Sep 26;4(9):e1000201. doi: 10.1371/journal.pgen.1000201 (PMC2533119; doi:10.1371/journal.pgen.1000201)
Supplement: Text S1 — Supplementary methods. (0.12 MB DOC) [file pgen.1000201.s005.doc]

# Plasmids and strains

Plasmids were checked by restriction digest and sequencing. *V. cholerae* strains were derived from the sequenced El Tor clinical isolate N16961 [1]. Mutations were made by allele exchange using derivative vectors of the R6Kγ-ori-based suicide vector, pDS132 [2] and using a strategy previously described [3]. For cloning purposes, *E. coli* strain Π1 (*pir*+) was used as a plasmid host. For conjugal transfer of plasmids to *V. cholerae* strains, *E. coli* β2163 was used as donor strains [4]. The integration of the genes of interest was confirmed by PCR. *E. coli* strains used for *in vivo* plasmid resolution assays were derived from FX223, a *rec*F, *xer*C::*Gm*^r^ (gentamicin), *xer*D::*Km*^r^ (kanamycin) derivative of AB1157. Inactivation of the RecF pathway was particularly helpful in getting clear resolution patterns, as it abolishes most homologous recombination on plasmids [5]. The related *xer*C and *xer*D genes were introduced in place of the *xer*C::*Gm*^r^ allele using derivative vectors of the pKO3 plasmid [6]. Derivatives of FX223 were rendered *fts*K_C_*^-^* by phage P1-mediated transduction of *fts*K1, a Δ*fts*K_C_::*Cm*^r^ allele that allows for the expression of a truncated form of the protein containing the N-terminal domain and two-thirds of the linker region [7]. *E. coli* strains used for growth competition assays were derived from LN2666. N, NLC^Ec^ and NLC^Hi^ have been previously described [8,9]. The *fts*K NLC^Vc^ alleles were cloned between two DNA segments corresponding to the upstream and downstream regions of the *fts*K ORF on the *E. coli* chromosome in an integration-excision vector derived from pLN135 [10]. A ΔLC allele tagged with a neo resistance gene (ΔLC-*Km*^r^) was first introduced into strain LN2666. The resulting strain, FX97, was then used for the ‘knock in’ of the other *fts*K alleles and for reference strain in growth competition assay. Correct integration of the alleles was further verified by PCR on genomic DNA.

**Plasmid and strain list**

Name Relevant genotype or features Reference

*E. coli* strains

BL834 *E. coli* BL21 pLysS cells for protein expression Lab stock

Π1 DH5α Δ*thy*A::(*erm^r^*-*pir116*) [4]

β2163 (F-) RP4-2-Tc::Mu Δ*dap*A::(*erm^r^*-*pir*) [4]

AB1157 *E. coli* K12 [11]

DS941 AB1157 *rec*F*143* *lac*I^q^ *lac*ZM15 [12]

FX223 DS941 Δ*xer*D^Ec^*::Km^r^* Δ*xer*C^Ec^*::Gm^r^* [9]

FX227 FX223 Δ*xer*C^Ec^*::*(*xer*C^Ec^- *xer*D^Ec^) , Δ*fts*K_C_::*Cm*^r^ [9]

FX229 FX223 Δ*xer*C^Ec^*::*(*xer*C^Vc^- *xer*D^Vc^) , Δ*fts*K_C_::*Cm*^r^ This study

MV5 FX223 Δ*xer*C^Ec^*::*(*xer*C^Vc^- *xer*D_YF_^Vc^) , Δ*fts*K_C_::*Cm*^r^ This study

MV6 FX223 Δ*xer*C^Ec^*::*(*xer*C_YF_^Vc^- *xer*D^Vc^) , Δ*fts*K_C_::*Cm*^r^ This study

MV7 FX223 Δ*xer*C^Ec^*::*(*xer*C_YF_^Vc^- *xer*D_YF_^Vc^) , Δ*fts*K_C_::*Cm*^r^ This study

LN2666 W1485 Str^r^ *, leu* , *thy*A , *deo*B or C , *sup*E , *rps*L [13]

FX97 LN2666 Δ*fts*KC^Ec^::*Km*^r^ [8]

FX98 FX97 Δ*fts*KC^Ec^::*Cm*^r^ [8]

FX99 FX97 Δ*fts*KC^Ec^::C^Ec^ [8]

FX102 FX97 Δ*fts*KC^Ec^::C^Hi^ [8]

MV1 FX97 Δ*fts*KC^Ec^::C^Vc^ This study

*V.cholerae* strains

CVC300 N16961 Str^r^ P_CP18_ (*ara*E-*Km^r^*) [3]

CVC301 N16961 Str^r^ P_CP18_ *ara*E [3]

MV25 CVC301 Δ*xer*C::*Sp*^r^ This study

MV26 CVC301 Δ*rec*A::*Km*^r^ This study

MV39 CVC301 Δ*dif*2::*Sp*^r^ This study

MV45 CVC301 Δ*dif*1::*Sp*^r^ This study

MV43 CVC301 Δ*xer*C::*Sp*^r^, Δ*rec*A This study

MV72 CVC301 Δ*dif*1::*Sp*^r^, Δ*rec*A This study

MV73 CVC301 Δ*dif*2:: *Sp*^r^, Δ*rec*A This study

Plasmids

pFX481 MBP-6His-XerD^Vc^ expression vector This study

pFX483 MBP-6His-XerC^Vc^ expression vector This study

pKO3 pSC101 *rep*Al(Ts), with *sac*B for allele exchange [6]

pFX381 pKO3 derivative for Δ*xer*C^Ec^*::*(*xer*C^Vc^- *xer*D^Vc^) This study

pMEV19 pKO3 derivative for Δ*xer*C^Ec^*::*(*xer*C^Vc^- *xer*D_YF_^Vc^) This study

pMEV20 pKO3 derivative for Δ*xer*C^Ec^*::*(*xer*C_YF_^Vc^- *xer*D^Vc^) This study

pMEV21 pKO3 derivative for Δ*xer*C^Ec^*::*(*xer*C_YF_^Vc^- *xer*D_YF_^Vc^) This study

pLN135 pSC101 *rep*Al(Ts), Δ*psi*, with *rps*L for allele exchange [10]

pFX399 pLN135 derivative for *fts*KC^Ec^::C^Vc^ This study

pDS132 R6Kγori , *mobRP4* , with *sac*B for allele exchange [2]

pMEV68 pDS132 derivative for Δ*rec*A This study

pMEV97 pDS132 derivative for Δ*rec*A::*Km*^r^ This study

pMEV70 pDS132 derivative for Δ*dif*1 This study

pMEV71 pDS132 derivative for Δ*dif*2 This study

pMEV72 pDS132 derivative for Δ*xer*C This study

pFX170 pBAD :: *fts*K^Ec^ under P_ara_ promoter [9]

pFX380 pBAD :: *fts*K^Vc^ under P_ara_ promoter This study

pFtsK^Ec^_50C[NRE]_ pBAD :: *fts*K^Ec^_50C[NRE]_ under P_ara_ promoter [14]

pMEV206 pBAD :: *fts*K^Vc^_[NRE]_ under P_ara_ promoter This study

pMEV43 pSC101::(*dif*Ec-*Cm^r^*-*dif*Ec) This study

pMEV173 pSC101::(*dif*1-*Cm^r^*-*dif*1) This study

pMEV170 pSC101::(*dif*2-*Cm^r^*-*dif*2) This study

pMEV174 pSC101::(*dif*12-*Cm^r^*-*dif*12) This study

pMEV175 pSC101::(*dif*13-*Cm^r^*-*dif*13) This study

pMEV169 pSC101::(*dif*14-*Cm^r^*-*dif*14) This study

pMEV39 pSC101::(*dif*15-*Cm^r^*-*dif*15) This study

pMEV176 pSC101::(*dif*23-*Cm^r^*-*dif*23) This study

pMEV172 pSC101::(*dif*1-*Cm^r^*-*dif*2) This study

pFX142(KOPS-0) pSC101::(*dif*Ec-*Km^r^*-*dif*Ec) [15]

pVS52(KOPS-2) pFX142 with non permissive KOPS [14]

# Growth competition assay

Very few cells carrying a dimer are expected to yield a viable progeny in the absence of CDR. Consequently, the proportion of cells that a mutant strain totally deficient in CDR fails to produce at each doubling time of its parent, which can be measured by growth competition experiments, gives a good estimation of the rate of chromosome dimer formation. This is true if the cell cycle of the mutant is not altered in the absence of dimers. This condition can be checked by measuring the growth defect of the mutated strain in a *rec*A context, in which no chromosome dimers can be formed by homologous recombination.

For growth competition of *E. coli* strains, the ratio of mutant and parental strains were determined every 20 generations (24 hours) by plating on chloramphenicol and kanamycin selective media. For growth competition of *V. cholerae* strains, the ratio of mutant and parental strains were determined by plating on spectinomycin and kanamycin selective media. The frequency of cells that a mutant strain fails to produce compared to its parent at each generation, f, equals 1-e^-k^, where k is the coefficient of the exponential describing the ratio of the mutant strain versus its parent, r, as a function of the number of generations, n (r = e^-kn^).

### *In vitro* Xer assays

G+A chemical cleavage of *dif* substrates were performed as described [16].

**Synthetic oligonucleotide list**

Name Sequence

B1

482 CGCGTTCTAGAAGTGCGcATTATGTATGTTATGTTAAATGAgatctgcg

483 cgcaGATCTCATTTAACATAACATACA

484 TAATgCGCACTTCTAGAAcgcg

T1

485 CGCGTTCTAGAAGTGCGcATTATGTATG

486 TTATGTTAAATGAgatctgcg

487 cgcaGATCTCATTTAACATAACATACATAATgCGCACTTCTAGAAcgcg

B2

497 CGCGTTCTAGAaatgcgcattacgtgcgttatgttaaatGAgatctgcg

498 CGCAGATCTCATTTAACATAACGCACG

499 TAATGCGCATTTCTAGAACGCG

T2

500 CGCGTTCTAGAaatgcgcattacgtgcg

501 ttatgttaaatGAgatctgcg

502 CGCAGATCTCATTTAACATAACGCACGTAATGCGCATTTCTAGAACGCG

**Data mining and phylogenic analysis**

Observation of the *dif* sequences from the γ-Proteobacteria revealed that, although the XerD binding site is well conserved, the XerC binding site and the central region are more variable. This is especially true for those species with multiple chromosomes. As BLAST is generally insensitive to the position of conservation in short nucleotide sequences, it is a poor tool for identifying addition *dif* sequences in species from the α- and β-Proteobacteria sub-domains. Highlighting this fact is that the published *dif* site of *Caulobacter crescentus* is not readily identifiable by BLAST search [17].  A more sensitive and adapted approach involved the use of Hidden Markov Models (HMMs), and the program HMMER. We used CLUSTALW [18] to format an alignment file of putative *dif* sequences from the larger chromosome of 27 γ-Proteobacteria (*Vibrio cholerae, Vibrio harveyi, Photobacterium profundum SS9, Vibrio parahaemolyticus, Vibrio vulnificus CMCP6, Vibrio vulnificus YJ016, Vibrio fischeri, Shewanella oneidensis, Shewanella putrefaciens CN32, Shigella flexneri58401, Salmonella typhimurium LT2, Salmonella enterica ATCC9150, Shigella boydii Sb227, Shigella dysenteriae Sd197, Escherichia coli K12, ShigellasonneiSs046, Pseudomonas entomophila, Pseudomonas mendocina, Pseudomonas putida F1, Pseudomonas syringae DC3000, Pseudomonas stutzeri A1501, Haemophilus influenzae RdKW20, Xanthomonas campestris 8004, Pseudomonas aeruginosa PAO1, Pseudomonas fluorescens Pf-5, Pseudoalteromonas haloplanktis, Pseudoalteromonas atlantica*). This alignment was used to generate the WebLogo in Figure 1B. It also served to generate a profile using the program HMMER [19]. This allowed us to analyze in position-specific details which of the 28 base pairs of *dif* were most strongly conserved. The Markov Models (HMMs) were then used to search FASTA files of chromosomal replicons from completely sequenced bacteria. In most cases, the result of HMMSEARCH included multiple equivalent (>1e-4) hits with a single putative *dif* site yielding a more significant score (<1e-5).

As the *dif* sequence is found at the junction of the two replichores, GC-skew data was generated for each chromosome using the Genome Skew Program [20]. In most cases the highest scoring hit from HMMER fell within 10 Kb of the GC-skew inflection point. We further confirm that the identified sequence was not found within a gene, as it is always found in intergenic regions in γ-Proteobacteria. Finally, we compared each sequence by hand to insure proper spacing of XerD and XerC binding region and the 6-bp central region.

The complete list of the *dif* sites we found is shown in Figure S2, S3 and S4. We could thus demonstrate that the divergence of the central regions of the chromosomal dimer resolution sites of all α-, β- and γ-Proteobacteria harboring multiple replicons is a constant attesting that chromosomal fusions are certainly detrimental in the wild: when paired together, *dif* sites carried within the same bacterium displayed a mean of 2.59 changes in the 6 bp central region (for a total of 27 pairs), compared to 0.17 changes when *dif* sites carried by bacteria harboring a single chromosomes were paired with *dif*^Ec^ (for a total of 29 pairs).

**References**

1. Heidelberg JF, Eisen JA, Nelson WC, Clayton RA, Gwinn ML, et al. (2000) DNA sequence of both chromosomes of the cholera pathogen Vibrio cholerae. Nature 406: 477-483.

2. Philippe N, Alcaraz JP, Coursange E, Geiselmann J, Schneider D (2004) Improvement of pCVD442, a suicide plasmid for gene allele exchange in bacteria. Plasmid 51: 246-255.

3. Srivastava P, Fekete RA, Chattoraj DK (2006) Segregation of the replication terminus of the two Vibrio cholerae chromosomes. J Bacteriol 188: 1060-1070.

4. Demarre G, Guerout AM, Matsumoto-Mashimo C, Rowe-Magnus DA, Marliere P, et al. (2005) A new family of mobilizable suicide plasmids based on broad host range R388 plasmid (IncW) and RP4 plasmid (IncPalpha) conjugative machineries and their cognate Escherichia coli host strains. Res Microbiol 156: 245-255.

5. Kolodner R, Fishel RA, Howard M (1985) Genetic recombination of bacterial plasmid DNA: effect of RecF pathway mutations on plasmid recombination in Escherichia coli. J Bacteriol 163: 1060-1066.

6. Link AJ, Phillips D, Church GM (1997) Methods for generating precise deletions and insertions in the genome of wild-type Escherichia coli: application to open reading frame characterization. J Bacteriol 179: 6228-6237.

7. Diez AA, Farewell A, Nannmark U, Nystrom T (1997) A mutation in the ftsK gene of Escherichia coli affects cell-cell separation, stationary-phase survival, stress adaptation, and expression of the gene encoding the stress protein UspA. J Bacteriol 179: 5878-5883.

8. Bigot S, Corre J, Louarn JM, Cornet F, Barre FX (2004) FtsK activities in Xer recombination, DNA mobilization and cell division involve overlapping and separate domains of the protein. Mol Microbiol 54: 876-886.

9. Yates J, Aroyo M, Sherratt DJ, Barre FX (2003) Species specificity in the activation of Xer recombination at dif by FtsK. Mol Microbiol 49: 241-249.

10. Cornet F, Louarn J, Patte J, Louarn JM (1996) Restriction of the activity of the recombination site dif to a small zone of the Escherichia coli chromosome. Genes Dev 10: 1152-1161.

11. Bachmann BJ (1972) Pedigrees of some mutant strains of Escherichia coli K-12. Bacteriol Rev 36: 525-557.

12. Summers DK, Sherratt DJ (1988) Resolution of ColE1 dimers requires a DNA sequence implicated in the three-dimensional organization of the cer site. EMBO J 7: 851-858.

13. Cornet F, Mortier I, Patte J, Louarn JM (1994) Plasmid pSC101 harbors a recombination site, psi, which is able to resolve plasmid multimers and to substitute for the analogous chromosomal Escherichia coli site dif. J Bacteriol 176: 3188-3195.

14. Sivanathan V, Allen MD, de Bekker C, Baker R, Arciszewska LK, et al. (2006) The FtsK gamma domain directs oriented DNA translocation by interacting with KOPS. Nat Struct Mol Biol 13: 965-972.

15. Aussel L, Barre FX, Aroyo M, Stasiak A, Stasiak AZ, et al. (2002) FtsK Is a DNA motor protein that activates chromosome dimer resolution by switching the catalytic state of the XerC and XerD recombinases. Cell 108: 195-205.

16. Sambrook J, Fritsch EF, Maniatis T (1989) Molecular cloning : a laboratory manual. Cold Spring Harbor, N.Y.: Cold Spring Harbor Laboratory Press.

17. Jensen RB (2006) Analysis of the terminus region of the Caulobacter crescentus chromosome and identification of the dif site. J Bacteriol 188: 6016-6019.

18. Thompson JD, Higgins DG, Gibson TJ (1994) CLUSTAL W: improving the sensitivity of progressive multiple sequence alignment through sequence weighting, position-specific gap penalties and weight matrix choice. Nucleic Acids Res 22: 4673-4680.

19. Durbin R (1998) Biological sequence analysis : probabalistic models of proteins and nucleic acids. Cambridge, UK New York: Cambridge University Press. xi, 356 p. p.

20. Edelstein M, Gehrke F, Hopf S, Jehl M, Oswald A, et al. (2003) Genome Skew. 1.0 ed.
